# NMR study of the structure and dynamics of the BRCT domain from the kinetochore protein KKT4

**DOI:** 10.1007/s12104-024-10163-9

**Published:** 2024-03-07

**Authors:** Patryk Ludzia, Hanako Hayashi, Timothy Robinson, Bungo Akiyoshi, Christina Redfield

**Affiliations:** 1 Department of Biochemistry, University of Oxford, South Parks Road, Oxford, OX1 3QU UK; 2 Wellcome Centre for Cell Biology, Institute of Cell Biology, School of Biological Sciences, University of Edinburgh, Michael Swann Building, Max Born Crescent, Edinburgh, EH9 3BF UK

**Keywords:** KKT4, Kinetoplastid, Kinetochore, BRCT, Trypanosomes, Phosphopeptide-binding, Hydrogen–deuterium exchange, NMR resonance assignments

## Abstract

KKT4 is a multi-domain kinetochore protein specific to kinetoplastids, such as *Trypanosoma brucei*. It lacks significant sequence similarity to known kinetochore proteins in other eukaryotes. Our recent X-ray structure of the C-terminal region of KKT4 shows that it has a tandem BRCT (BRCA1 C Terminus) domain fold with a sulfate ion bound in a typical binding site for a phosphorylated serine or threonine. Here we present the ^1^H, ^13^C and ^15^N resonance assignments for the BRCT domain of KKT4 (KKT4^463–645^) from *T. brucei*. We show that the BRCT domain can bind phosphate ions in solution using residues involved in sulfate ion binding in the X-ray structure. We have used these assignments to characterise the secondary structure and backbone dynamics of the BRCT domain in solution. Mutating the residues involved in phosphate ion binding in *T. brucei* KKT4 BRCT results in growth defects confirming the importance of the BRCT phosphopeptide-binding activity in vivo. These results may facilitate rational drug design efforts in the future to combat diseases caused by kinetoplastid parasites.

**Supplementary Information:**

The online version contains supplementary material available at 10.1007/s12104-024-10163-9.

## Biological context

Every time a cell divides, it must accurately replicate and distribute its genetic material to daughter cells. Failure to do so leads to genetic abnormalities that can result in cancer or cell death. Chromosome segregation is therefore an essential process for ensuring the survival of all organisms (McIntosh 2016). A structure that plays key roles in the process of chromosome segregation is the kinetochore, a multiprotein assembly that connects chromosomal DNA and spindle microtubules during mitosis (Brinkley and Stubblefield 1966; Cheeseman 2014). Many kinetochore components are widely conserved among eukaryotes, especially CENP-A and Ndc80 that bind DNA and microtubules, respectively (Meraldi et al. 2006; Cheeseman and Desai 2008; Santaguida and Musacchio 2009; Biggins 2013; van Hooff et al. 2017).

Interestingly, none of the conventional kinetochore proteins has been found in kinetoplastids, an evolutionarily divergent group of unicellular flagellated eukaryotes including parasitic trypanosomatids (e.g. *Trypanosoma brucei, Trypanosoma cruzi,* and *Leishmania* species) (Berriman et al. 2005). Instead, a group of unique kinetochore proteins (KKT1–25 and KKIP1–12) has been identified in *T. brucei*; these proteins have no significant sequence similarity to conventional kinetochore proteins found in other eukaryotes (Akiyoshi and Gull 2014; Nerusheva and Akiyoshi 2016; D'Archivio and Wickstead 2017; Nerusheva et al. 2019; Brusini et al. 2021).

**Fig. 1:**
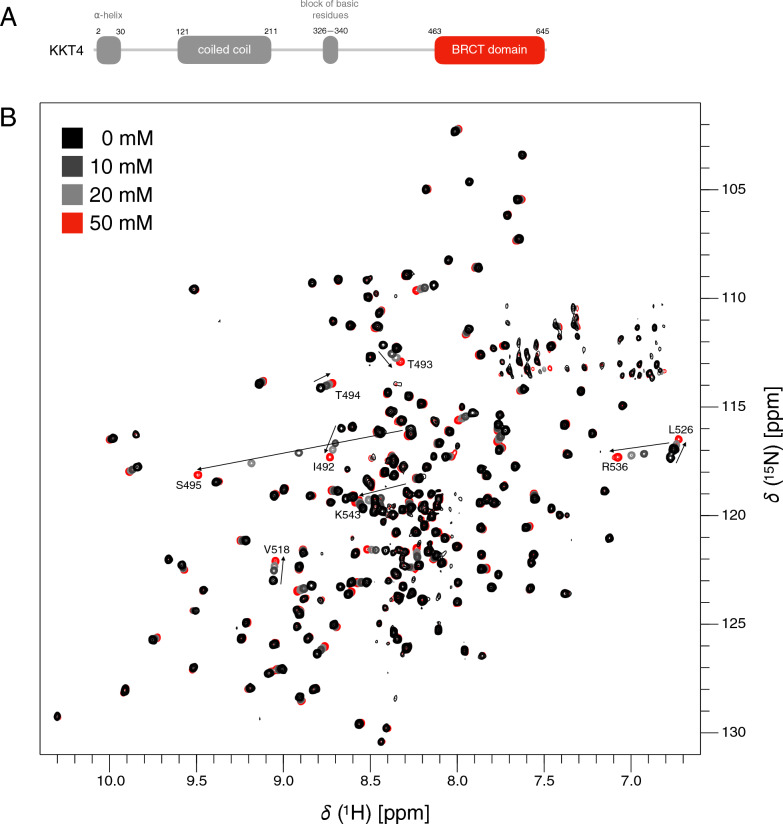
**A** Schematic representation of the domain organisation of KKT4. **B** Overlay of 750 MHz ^1^H-^15^N BEST TROSY spectra of ^15^N-KKT4^463–645^ (black) and ^15^N-KKT4^463–645^ in the presence of 10 (dark grey), 20 (light grey) and 50 (red) mM sodium phosphate. The addition of phosphate ion resulted in chemical shift changes for several residues, indicating the binding of phosphate to KKT4 BRCT. The residues that shifted the most are labeled. These residues are located in the BRCT1 subdomain close to the sulphate ion binding site identified in the X-ray structure (Fig. 2A)

KKT4 is an essential kinetochore component and has microtubule-binding activity (Llauro et al. 2018). We recently reported the X-ray structure of the KKT4 C-terminal region (see Fig. 1A for the domain organization of KKT4), showing that it has a tandem BRCT (BRCA1 C Terminus) domain fold (Ludzia et al. 2021). Interestingly, no kinetochore protein found in other eukaryotes contains a BRCT domain (Musacchio and Desai 2017). Therefore, the function of this domain in KKT4 cannot be inferred from studies of other eukaryotic kinetochore proteins. Our initial analysis suggested that the BRCT domain in KKT4 is involved in phosphopeptide binding (Ludzia et al. 2021). Here we present ^1^H, ^13^C and ^15^N resonance assignments for the KKT4 BRCT domain (KKT4^463–645^, MW 19.9 kDa) from *T. brucei*. These NMR assignments provide a starting point for detailed investigations of the structure, dynamics, interactions and function of the KKT4 BRCT domain in solution.

## Methods and experiments

### Protein expression and purification

The KKT4^463—645^ used in this study was amplified from *T. brucei* genomic DNA that was cloned into the pNIC28-Bsa4 expression vector using a ligation-independent cloning method (Gileadi et al. 2008, Ludzia et al. 2021). *E. coli* BL21(DE3) cells were transformed with ~ 100 ng of plasmid DNA and were inoculated into 40 ml of 2xTY media containing 50 µg/ml kanamycin. Cells were grown at 37 °C overnight. The next day, cells were spun down at 3,400 g for 10 min and resuspended in 40 ml of M9 minimal medium containing 50 µg/ml kanamycin, 1 g/L ^15^NH_4_Cl and 4 g/L [^13^C]-*D*-glucose (CIL) as the sole nitrogen and carbon sources. Next, the resuspended culture was inoculated into 1L of M9 minimal medium supplemented with 1 g/L ^15^NH_4_Cl, 4 g/L [^13^C]-*D*-glucose and 50 µg/ml kanamycin. Cells were grown at 37 °C to an OD_600_ of 0.9–1.0 and protein expression was induced using 0.4 mM IPTG. Protein expression was continued overnight at 16 °C with shaking (200 rpm). Purification of the BRCT domain using the following protocol results in ~ 1 mg of pure protein from 1 L of bacterial culture. The ^15^N-labelled protein was produced using the same protocol except that unlabeled glucose was used.

Cells were pelleted at 3400 g at 4 °C and resuspended in lysis buffer (50 mM sodium phosphate pH 7.5, 500 mM NaCl, and 10% glycerol) supplemented with protease inhibitors (20 μg/ml leupeptin, 20 μg/ml pepstatin, 20 μg/ml E-64, 0.4 mM PMSF), benzonase nuclease (500 U/1L culture), and 0.5 mM TCEP. All subsequent extraction steps were performed at 4 °C. Cell lysis was facilitated by mechanical cell disruption (French press, 1 passage at 20,000 psi). Lysed cells were spun at 48,000 g for 30 min and the supernatant was loaded on a gravity column with TALON beads (Takara Clontech) pre-equilibrated in lysis buffer. After loading, the beads were washed extensively with lysis buffer and proteins were eluted with elution buffer (50 mM sodium phosphate pH 7.5, 500 mM NaCl, 10% glycerol, 250 mM imidazole, 0.5 mM TCEP). To remove the N-terminal 6xHis tag, the protein was incubated with TEV protease in a 1:50 w/w ratio and dialysed overnight into 25 mM sodium phosphate pH 7.5, 250 mM NaCl, 5% glycerol, 5 mM imidazole and 0.5 mM TCEP. Note that S461 and M462 at the protein N-terminus are the result of the TEV cleavage and they precede the first residue of the BRCT domain (S463). To increase the sample purity and remove the cleaved 6xHis tag, the protein solution was re-loaded on the TALON beads equilibrated in the dialysis buffer. The collected flow-through was concentrated and loaded on a gel filtration column SD200 16/60 (GE Healthcare) to purify further and buffer exchange the sample into 50 mM sodium phosphate pH 7.0, 100 mM NaCl and 0.5 mM TCEP. The fractions containing the KKT4^463–645^ BRCT domain were pooled, concentrated to 500 μM and flash-frozen in liquid nitrogen. Expression and purification of the KKT4^463–645^ K543A mutant (pBA2264) for crystallisation was carried out as previously described for the wild-type KKT4^463–645^ (Ludzia et al. 2021).

### NMR spectroscopy

Initially, a ^1^H-^15^N BEST TROSY spectrum was collected at 20°C for 100 μM KKT4^463–645^ in 25 mM HEPES pH 7.1, 150 mM NaCl and 0.5 mM TCEP. To test for the interaction of KKT4^463–645^ with phosphate ion, stepwise additions of sodium phosphate up to a concentration of 50 mM were made and ^1^H-^15^N BEST TROSY spectra were collected.

^15^N or ^13^C/^15^N-labelled samples of KKT4^463–645^ were used for resonance assignment using standard triple-resonance protocols (Redfield 2015). All samples used for the data collection contained ~ 500 μM BRCT domain in a 50 mM sodium phosphate buffer at pH 7.0 with 100 mM NaCl, 0.5 mM TCEP and 5% D_2_O. The NMR experiments were carried out at 20 °C using either a 750 or a 950 MHz spectrometer; both spectrometers are equipped with Oxford Instruments Company magnets, Bruker Avance III HD consoles and 5 mm TCI CryoProbes. Salt-tolerant Bruker shaped NMR tubes were used for data collection.

Backbone resonance assignments for KKT4^463–645^ were obtained using 2D ^1^H-^15^N BEST-TROSY and ^1^H-^15^N HSQC experiments and 3D experiments including ^15^N-edited NOESY-HSQC, and TOCSY-HSQC and BEST-TROSY versions (Schulte-Herbruggen and Sorensen 2000; Lescop et al. 2007) of HNCA, HNCACB, HN(CO)CACB, HNCO and HN(CA)CO and a 3D HBHA(CBCACO)NH. Side chain resonance assignments for KKT4^463–645^ were obtained using 2D ^1^H-^13^C HSQC experiments and 3D experiments including ^15^N-edited TOCSY-HSQC, (H)CC(CO)NH, H(CCCO)NH, HCCH-TOCSY, (H)CCH-TOCSY, HCCH-COSY, (H)CCH-COSY and ^13^C-edited NOESY-HSQC. All experiments except for the HCCH-TOCSY were collected at 750 MHz. All 3D experiments were collected with 25% non-uniform sampling in the two indirect dimensions using standard Bruker sampling schedules. 2D NMR data were processed using NMRPipe (Delaglio et al. 1995) and 3D NUS data were processed with the hmsIST software (Hyberts et al. 2012) and NMRPipe. Spectra were analysed and assignments recorded using CCPN Analysis version 2.5 (Vranken et al. 2005). ^1^H and ^13^C chemical shifts were referenced using DSS and ^15^N chemical shifts were referenced indirectly. Details of the specific experiments and sample conditions can be found in the BMRB deposition file (BMRB 51542). ^1^H, ^13^C and ^15^N chemical shifts of KKT4 BRCT were analysed using TALOS-N (Shen and Bax 2013) to predict secondary structure propensities.

### Hydrogen–deuterium exchange

Slowly exchanging amides were identified from ^1^H-^15^N BEST-TROSY spectra collected 15 min, 24 h and 48 h after a sample of 450 μM KKT4^463–645^ was buffer exchanged into 50 mM sodium phosphate D_2_O buffer containing 100 mM NaCl and 0.5 mM TCEP at pH 7.1 using a 0.5 ml Zeba Spin (ThermoFisher) 7kDa cutoff desalting column. The extent of hydrogen–deuterium exchange of each amide was calculated from peak intensities.

### Backbone dynamics

The {^1^H}-^15^N heteronuclear NOE was measured at 20℃ using the TROSY-based heteronuclear NOE experiment recorded with and without ^1^H saturation for 4s at 750 MHz (Zhu et al. 2000). The {^1^H}-^15^N NOE was calculated as the ratio of the peak intensities in the spectra recorded with and without ^1^H saturation. {^1^H}-^15^N heteronuclear NOE ratios were not calculated for overlapping peaks. Peak heights were determined using CCPN Analysis (Vranken et al. 2005). Uncertainties in the {^1^H}-^15^N NOE values were estimated from 500 Monte Carlo simulations using the baseline noise as a measurement of the error in the peak heights.

### Residual Dipolar Couplings (RDCs)

Isotropic ^1^H^N^-^15^N splittings were measured for a 330 μM sample of ^15^N-labeled KKT4^463–645^. Partial alignment of KKT4^463–645^ was achieved using C12E6/*n*-hexanol liquid crystals prepared as described by Rückert and Otting (Rückert and Otting 2000). Briefly, a 15% C12E6/*n-* hexanol stock solution was prepared in phosphate buffer (50 mM sodium phosphate, 100 mM NaCl). 100 μl of this stock solution was added to 200 μl of the 330 μM ^15^N-labelled KKT4^463–645^ to achieve a final concentration of 5% C12E6/*n-*hexanol. ^1^H^N^-^15^N splittings for both samples were measured using BEST TROSY and semi-BEST TROSY experiments at 20℃ (Schulte-Herbruggen and Sorensen 2000; Lescop et al. 2007). RDCs were measured as the difference between the splitting observed in the isotropic and aligned data sets. Three measurements were taken in each experiment and the average RDC value was calculated.

The principal components and orientation of the molecular alignment tensor were fitted to minimise the χ^2^ between the experimental and calculated RDCs using the KKT4^463–645^ X-ray coordinates (PDB:6ZPK) to which ^1^H had been added using X-PLOR version 3.8 (Brünger 1992). Residues with {^1^H}-^15^N heteronuclear NOE values of less than 0.7 were excluded from the fitting procedure. Q values were calculated to assess the quality of the fits between experimental and calculated RDCs using the method of Cornilescu and co-workers (Cornilescu et al. 1998).

### Crystallisation, diffraction data collection and structure determination

Crystals of *T. brucei* KKT4^463–645^ K543A (24 mg/ml) were grown at 4℃ in the JCSG + crystallisation screen (Hampton Research) solution containing 0.2 M sodium thiocyanate and 20% w/v PEG3350. Crystals were briefly transferred into mother liquor prepared with addition of 23% glycerol prior to flash-cooling by plunging into liquid nitrogen.

X-ray diffraction data from *T. brucei* KKT4^463–645^ K543A crystals were collected at the I03 beamline at the Diamond Light Source (Harwell, UK). The structure was solved using PHASER (McCoy et al. 2007) and the wild-type KKT4^463–645^ crystal structure as a model (PDB: 6ZPK), followed by initial model building with BUCCANEER (Cowtan 2006). Further model building and refinement were completed using COOT (Emsley et al. 2010) and PHENIX (Liebschner et al. 2019). The dataset used for the final refinement was scaled to the high-resolution limit of 1.8 Å and processed using anisotropic scaling (Strong et al. 2006). The final refinement statistics are summarised in Supporting Table 1. Protein coordinates have been deposited in the RCSB Protein Data Bank (http://www.rcsb.org/) with the accession code PDB: 7QRO. All structure figures were prepared using PyMOL (DeLano 2002).

### Trypanosome cell lines and fluorescence microscopy

All cell lines used in this study were derived from *Trypanosoma brucei* SmOxP927 procyclic form cells (TREU 927/4 expressing T7 RNA polymerase and the tetracycline repressor to allow inducible expression) (Poon et al. 2012). Cells were grown at 28°C in SDM-79 medium supplemented with 10% heat-inactivated fetal calf serum and 7.5 μg/ml hemin (Brun and Schonenberger 1979). All DNA primers used in this study are listed in Supporting Table 2. All constructs were sequence verified. To make pBA835 (doxycycline-inducible expression of GFP-NLS-KKT4^344–645^), the KKT4^344–645^ fragment was amplified from genomic DNA with BA1363/BA856 and cloned into pBA310 (Nerusheva and Akiyoshi 2016) cleaved with PacI and AscI. The K543A mutation (pBA1777) and T493A/T494A mutations (pBA1847) were introduced into pBA835 by Quickchange PCR using BA1732/BA1733 and BA2300/BA2301 primer pairs, respectively. These plasmids were cleaved with NotI, transfected by electroporation into 177 bp repeats on minichromosomes in BAP308 (SmOxP927 with a kinetochore marker, tdTomato-KKT2 (Nerusheva and Akiyoshi 2016)), and selected by the addition of 5 μg/ml phleomycin to make BAP907, BAP909, and BAP1611. Endogenous tagging of KKT4 with C-terminal YFP was performed using pBA1518 (Llauro et al. 2018). The K543A mutation (pBA1536) and T493A/T494A mutations (pBA2104) were introduced into pBA1518 by Quickchange PCR using BA1732/BA1733 and BA2300/BA2301, respectively. These plasmids linearised by NotI were transfected to trypanosomes by electroporation into an endogenous locus. Transfected cells were selected by the addition of 25 μg/ml hygromycin, cloned by dispensing dilutions into 96-well plates, and screened by PCR and DNA sequencing (to make BAP1256: WT, BAP1908: K543A, and BAP1907: T493A, T494A). pBA1398 (the RNAi construct targeting the 3’UTR of KKT4 (Llauro et al. 2018)) cleaved with NotI was then transfected into these cell lines to make BAP1450, BAP1940, and BAP1938. Cells were fixed with 4% paraformaldehyde as previously described (Nerusheva and Akiyoshi 2016). Images were captured at room temperature on a DeltaVision fluorescence microscope (Applied Precision) installed with softWoRx version 5.5 housed in the Oxford Micron facility. Fluorescent images were captured with a CoolSNAP HQ camera using 60 × objective lenses (1.42 NA) (~ 16 z sections at 0.2 μm steps). Images were processed in ImageJ (Schneider et al. 2012).

## Extent of assignments and data deposition

### Resonance assignment

**Fig. 2:**
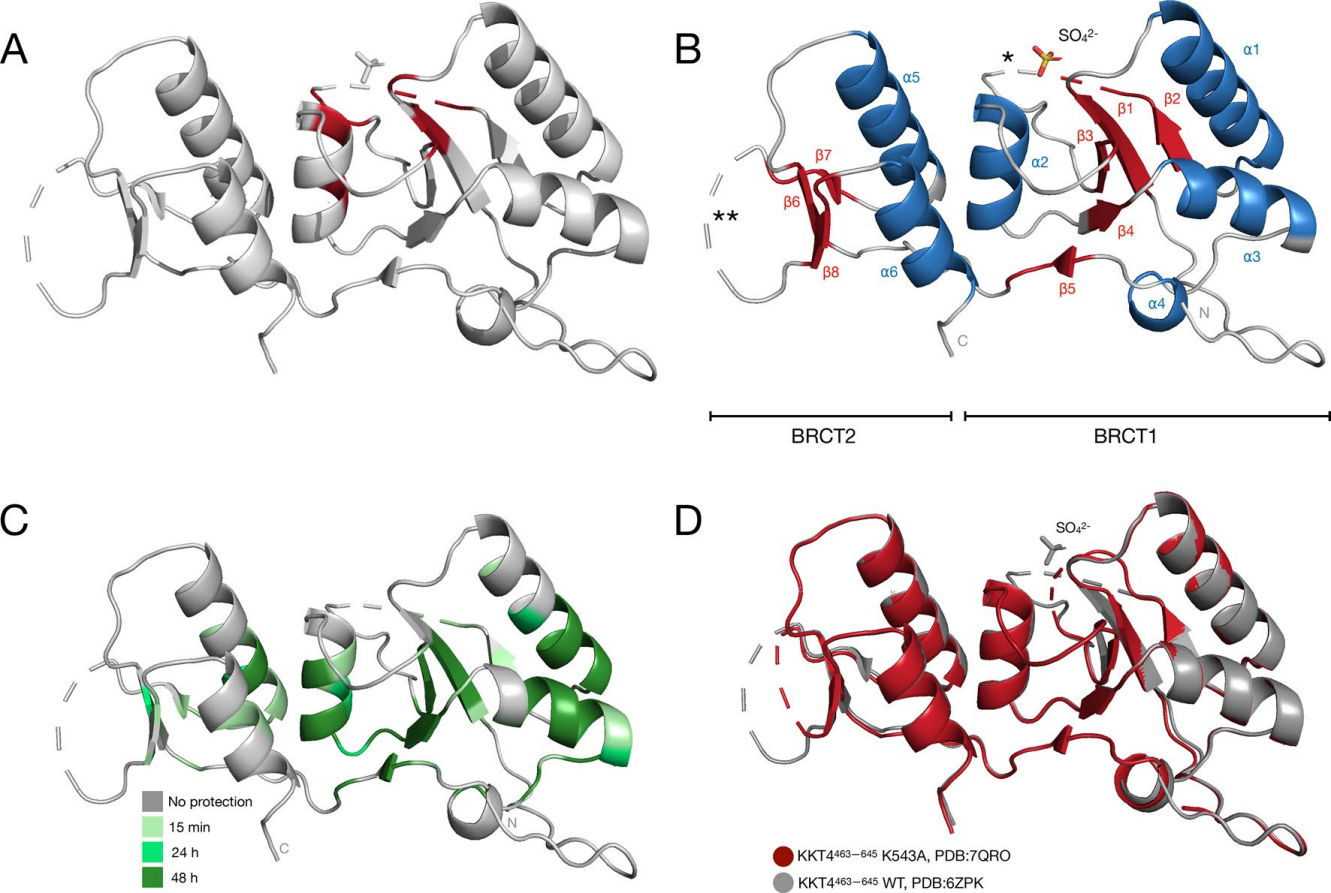
**A** Residues for which the largest chemical shift changes were observed (> 0.1 ppm) upon addition of 50 mM sodium phosphate are shown in red on the cartoon representation of the KKT4 BRCT crystal structure (PDB: 6ZPK). The highlighted residues are located in BRCT1 and correspond to residues around the sulfate binding site observed in the X-ray structure. **B** The secondary structure elements identified using TALOS-N (Fig. 4B) are highlighted in the cartoon representation of the crystal structure of KKT4 BRCT. The α-helices and β-strands are coloured in blue and red, respectively, and are labelled α1-α6 and β1-β8. The asterisks indicate the loop regions for which no electron density is observed in the crystal structure. The two subdomains, BRCT1 and BRCT2 are indicated. The first residue at the N-terminus showing electron density is residue 474. **C** The cartoon representation of the crystal structure of KKT4 BRCT is coloured to show protected amides observed in the hydrogen–deuterium exchange experiment. Residues giving observable peaks in the BEST TROSY after 15 min are shown in light green, those observed after 24 h are shown in mid-green and those observed after 48 h are shown in dark green. **D** Superposition of the cartoon representation of the wild-type KKT4 BRCT domain (grey, PDB: 6ZPK) with the K543A mutant of KKT4 BRCT (red, 7QRO). The structures show an excellent structural match, indicating that the K543A mutation did not perturb the BRCT structure

**Fig. 3:**
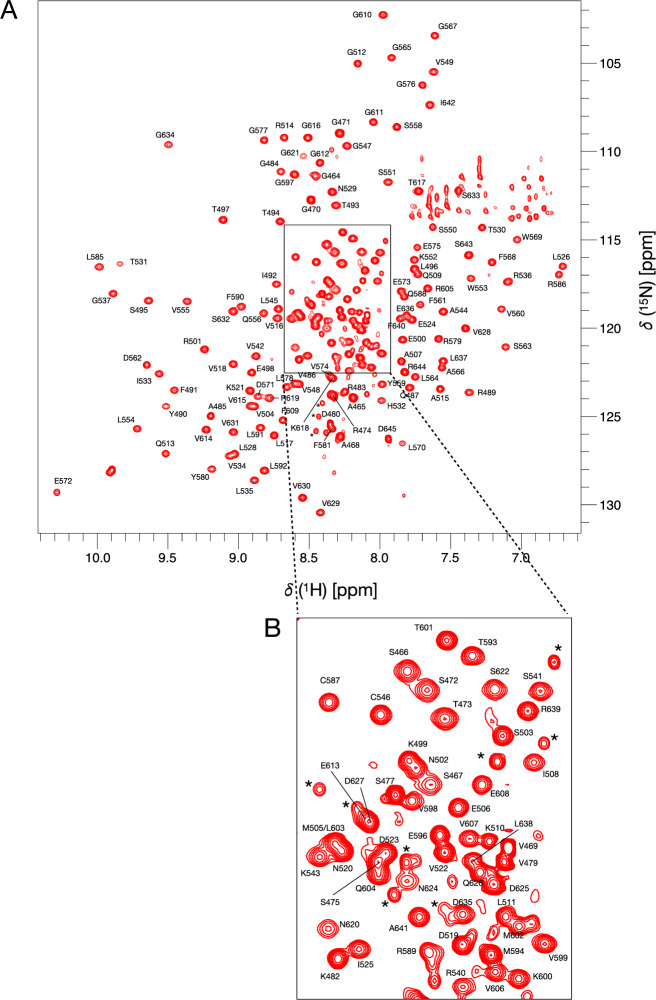
**A** 750 MHz ^1^H-^15^N BEST-TROSY spectrum of KKT4 BRCT in 50 mM sodium phosphate, 100 mM NaCl and 0.5 mM TCEP (95% H_2_O/5% D_2_O), at pH 7.0, 20℃. The peak assignments for backbone amides of KKT4 BRCT are annotated. Peaks in the region of 111–114 ppm and upfield of ~ 7.6 ppm are artefacts in the BEST-TROSY arising from incomplete cancellation of signal from the side chain amides of Asn and Gln; this region of a ^1^H-^15^N HSQC spectrum showing the Asn and Gln side chain amide assignments is shown in Supporting Fig. 1. **B** An expansion of the crowded area of the spectrum outlined in A with the black box. Peaks indicated with a * correspond to minor conformations of residues that are located close in sequence to proline residues in the disordered N-terminal region of the protein (S475-P476-S477-P478-V479-D480-P481-K482)

In the X-ray structure of KKT4 BRCT, a sulphate ion, introduced from the crystallisation buffer condition, was observed in the phosphopeptide binding pocket conserved in BRCT domains (Ludzia et al. 2021). In order to test whether the KKT4 BRCT domain binds phosphate in this pocket, NMR spectra were collected with increasing phosphate ion concentration (0 to 50 mM) (Fig. 1B). Large chemical shift changes were observed for residues subsequently assigned to I492, T493, S495, V518, L526, R536 and K543, all located in the BRCT1 subdomain (Fig. 2A). The X-ray structure shows that T493, S495 and K543 coordinate the sulphate ion (Ludzia et al. 2021). Our NMR data therefore confirm that KKT4 BRCT binds phosphate in the proposed phosphopeptide-binding pocket. Several peaks in the spectrum showed increased intensity in the presence of phosphate ion so the NMR resonance assignments were conducted in a 50 mM phosphate buffer (Fig. 3).

The ^1^H-^15^N BEST-TROSY spectrum of the KKT4 BRCT domain shows approximately 200 well-dispersed peaks characteristic of a structured protein (Fig. 3A, B); however, there are more peaks than expected for a protein containing 171 non-proline residues. Many of the additional peaks, indicated by * in Fig. 3B, have been assigned to residues that are close in the sequence to proline residues that undergo cis/trans isomerisation; these residues are mainly located near the N-terminus of KKT4 BRCT. To obtain backbone and side chain assignments, triple resonance data were acquired at pH 7.0 and 20℃. ^1^H^N^ and ^15^N backbone resonances for 169 of the 171 non-proline residues of KKT4 BRCT were assigned, corresponding to 98.8% of ^1^H^N^ and non-proline ^15^N chemical shifts. Assignments are only missing for S463 and S623; these residues, which are located in the unstructured N-terminus and in a loop, may be missing due to fast exchange of the ^1^H^N^ with solvent at pH 7.0. Backbone resonance assignments were also obtained for 97.8% of ^13^Cα, 96.2% of ^13^C' and 89.6% of ^1^Hα. Side chain ^1^H and ^13^C resonance assignments were obtained for 78.5%/96.4% of ^1^Hβ/^13^Cβ, for 66.7%/68.3% of ^1^Hγ /^13^Cγ, for 47.7%/39.5% of ^1^Hδ /^13^Cδ and for 25.8%/15.4% of ^1^Hε/^13^Cε. Many of the missing assignments correspond to repeats of two or three prolines in the sequence (P538/P539 and P582/P583/P584). The ^1^Hδ/ε and ^15^Nδ/ε side chain resonances of all 5 Asn and 6 of the 7 Gln residues have also been assigned (Supporting Fig.1). The ^1^H, ^13^C and ^15^N chemical shift assignments presented here for KKT4 BRCT have been deposited in the BioMagResBank (http://www.bmrb.wisc.edu) under the accession number 51542.

### Analysis of secondary structure

**Fig. 4:**
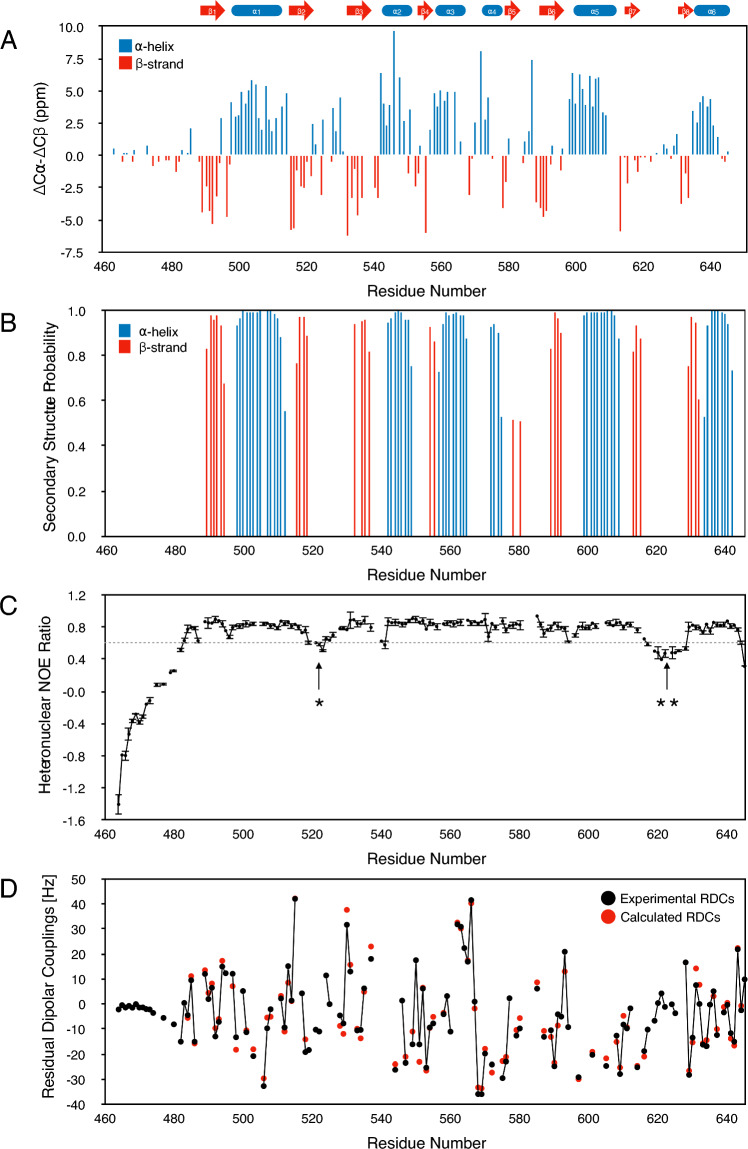
**A** The difference between the Cα and Cβ secondary shifts (ΔCα-ΔCβ) are plotted against the sequence of KKT4 BRCT. Negative and positive ΔCα-ΔCβ values are indicative of β-strand and α-helical secondary structure, respectively. A cartoon representation of the secondary structure elements identified in the crystal structure of KKT4 BRCT is shown above this panel. **B** TALOS-N secondary structure analysis of KKT4 BRCT. TALOS-N predicts 6 α-helices and 8 β-strands. The secondary structure elements for which the probability was lower than 0.5 are not shown. **C** The {^1^H}-^15^N heteronuclear NOE ratios are plotted against the sequence of KKT4 BRCT. Most residues display hetNOE ratios > 0.7, indicating a rigid conformation of the BRCT domain backbone. The regions with higher flexibility (ratios < 0.7) correspond to 464–483 near the N-terminus, 519–525 (*) and 616–628 (**). **D** Experimental residual dipolar couplings (RDCs) (black circles) are plotted as a function of the sequence of KKT4 BRCT. The near zero values for residues at the N-terminus are consistent with the flexibility of this region identified in the hetNOE experiment. RDCs calculated with a single D_a_ and R value but slightly different orientations of the alignment tensor are plotted for the 81 residues used in the fitting process (red circles) (see also Supporting Fig. 2)

The chemical shifts obtained for KKT4 BRCT have been analysed using the Cα-Cβ secondary shift difference (Spera & Bax 1991; Metzler et al. 1993) and TALOS-N (Shen and Bax 2013) to identify regions of secondary structure in solution (Fig. 4A, B); KKT4 BRCT contains 6 α-helices and 8 β-strands. These predicted secondary structure elements are in excellent agreement with the elements observed in the crystal structure (Fig. 2B). Moreover, regions for which no electron density was observed in the crystal structure are identified as ‘coil’ by TALOS-N.

Hydrogen–deuterium exchange experiments were also used to identify stable hydrogen-bonded structure in KKT4 BRCT (Fig. 2C). A sample was buffer exchanged into a D_2_O buffer and a BEST TROSY spectrum was collected within 15 min. Peaks from 70 residues were observed in this spectrum. These residues are mostly located in α-helices or β-strands but some correspond to loop regions (Fig. 2B). Further spectra were collected after 24 and 48 h; 26 of the 70 residues observed in the first spectrum have exchanged completely within 24 h. In general, amides in the larger BRCT1 subdomain are more protected than those in the smaller BRCT2. A group of 35 residues are still observed in the BEST TROSY spectrum after 48 h at pH 7.1; the amides of all of these residues, except Y580, are involved in hydrogen bonds in the crystal structure of KKT4 BRCT. Y580 is located in the β5 strand which is predicted by TALOS-N to be longer in solution than the β-strand observed in the crystal structure.

### Heteronuclear NOE

To study the fast timescale backbone dynamics of KKT4 BRCT, the {^1^H}-^15^N heteronuclear NOE experiment was performed (Fig. 4C). Most residues have hetNOE intensity ratios of > 0.7, indicating a rigid backbone. The regions between 519–525 and 616–628 display lower hetNOE ratios (< 0.7), indicating higher flexibility. This observation is consistent with the lack of electron density in the crystal structure for residues between 519–523 and 617–625. The N-terminal residues (464–483) of the BRCT domain show the most pronounced flexibility; this is consistent with the absence of electron density for residues 463–473 and the lack of protection for these residues in the hydrogen–deuterium exchange experiment (Fig. 2C). The two subdomains are connected by a very short linker involving residues 585–587; these residues do not show a reduced hetNOE suggesting that the interdomain linker is not flexible on a fast timescale. In addition, peaks for residues 585 and 586 are observed in the first H/D exchange spectrum, collected after 15 min (Fig. 2C).

### Residual dipolar couplings

RDCs were measured for the BRCT domain in 5% C12E6/hexanol (Fig. 4D). The RDCs range from values of -35.8 Hz to + 42.0 Hz indicating strong alignment as expected from the cylindrical shape of the BRCT domain (Fig. 2). Residues near the N-terminus of the BRCT domain have experimental RDCs close to zero; this is consistent with the low hetNOE values for these residues which are typical of a disordered region of the polypeptide chain. The molecular alignment tensor in the X-ray structure was fitted to minimise the χ^2^ between the experimental and calculated RDCs; this was done initially for each of the two individual domains (BRCT1 and BRCT2). Residues with hetNOE values of less than 0.7 were excluded from the analysis. Good agreement between the experimental and calculated RDCs is obtained for the individual domains with Q values of 0.17 and 0.19 for 52 and 29 residues, respectively, for the BRCT1 and BRCT2 domains. If the RDCs for the two domains are combined and fitted to the X-ray structure then the Q value increases to 0.24 for the 81 residues. In order to test if the relative orientations of the two domains in solution might differ from that seen in the X-ray structure, another fit was carried out in which a single D_a_ and R value is used for the two domains but the orientation of the alignment tensor within the two domains is allowed to differ. In this fit, an overall Q value of 0.18 was obtained (0.17 for BRCT1 and 0.19 for BRCT2) (Supporting Fig. 2). This improved agreement is statistically significant (F-value 8.61, probability of statistical significance 0.9999) and suggests that the structure in solution may differ slightly from the X-ray structure; the angle θ that defines the orientation of the principal component of the alignment tensor differs by ~ 7° for the two domains.

### T493, T494 and K543 located within the KKT4 BRCT domain are essential for *Trypanosoma brucei* viability

**Fig. 5:**
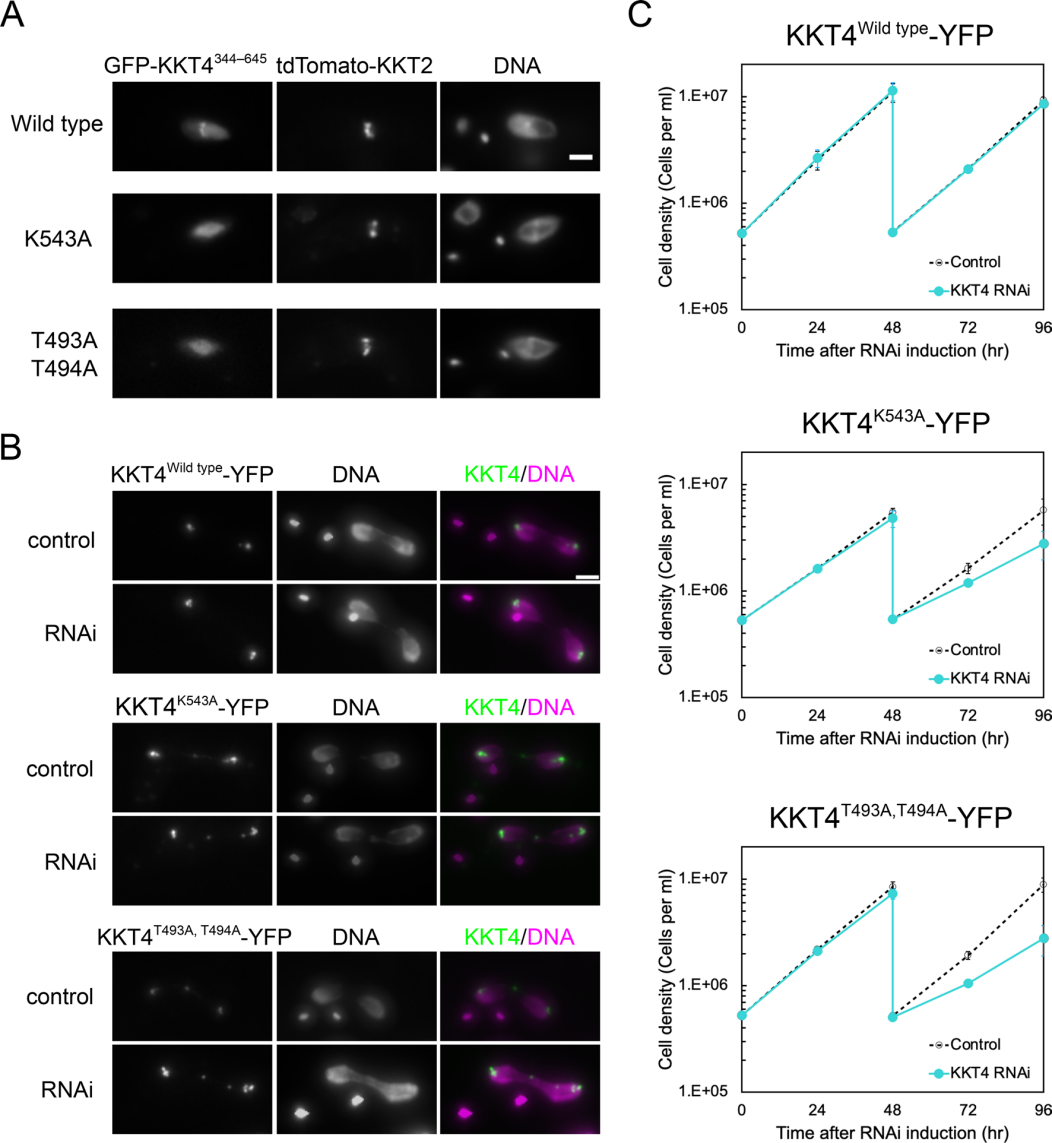
**A** Fluorescence microscopy images showing the localisation of exogenously expressed KKT4^344–645^ wild type, KKT4^344–645^ K543A, and KKT4^344–645^ T493A/T494A in metaphase trypanosome cells. Note that kinetochore localisation was observed only for the wild-type protein (n > 20 cells each). Expression of GFP-KKT4^344–645^ proteins was induced with 0.01 μg/ml doxycycline for 1 day. tdTomato-KKT2 was used as a kinetochore marker. Bar, 2 μm. **B** Fluorescence microscopy images showing the localisation of C-terminally YFP-tagged full-length KKT4^Wild type^, KKT4^K543A^ and KKT4^T493A,T494A^ in anaphase cells. Expression of the KKT4 RNAi construct that targets its 3’UTR and depletes untagged KKT4 copy was induced with 0.1 μg/ml doxycycline for 30 h. These mutants localise at kinetochores even after induction of KKT4 RNAi. Bar, 2 μm. **C** Growth curves of trypanosome cells expressing KKT4^Wild type^, KKT4^K543A^, or KKT4^T493A,T494A^, which was monitored over 96 h after the induction of KKT4 RNAi with 0.1 μg/ml doxycycline (n = 2). Cells were diluted at 48 h. Expression of the wild-type KKT4, not the mutants, rescued the growth defect caused by KKT4 RNAi, showing the importance of T493, T494 and K543 for the KKT4 function

While expressing various KKT4 fragments in trypanosomes in vivo, we found that the C-terminal region of KKT4 (KKT4^344–645^, containing a disordered linker and the BRCT domain) can localise to the kinetochore when ectopically expressed in trypanosomes using a doxycycline-induction system (Fig. 5A). To test the importance of phosphopeptide-binding activity of the KKT4 BRCT domain in the observed kinetochore localisation, we mutated residues located in the phosphopeptide-binding site. Our previous work has shown that the K543A mutant of the KKT4 BRCT domain bound a KKT8-derived phosphopeptide with a substantially lower affinity than the wild-type protein in vitro (Ludzia et al. 2021). We also mutated T493 and T494 that showed large shifts upon phosphate ion binding (Fig. 1B). Interestingly, both K543A and T493A/T494A mutants of KKT4^344–645^ failed to localise at the kinetochore (Fig. 5A). This result implies that KKT4^344–645^ localises at kinetochores via the phosphorylation-dependent protein–protein interaction by the BRCT domain and that the binding partner of the BRCT domain is localised at the kinetochore. To confirm that the defects observed with the K543A mutation were not due to an altered KKT4 BRCT structure, the crystal structure of KKT4^463–645^ K543A was determined to 1.8 Å resolution (Supporting Table 1, Fig. 2D). The wild-type and K543A structures overlay with an RMSD of 0.14 Å (for 919 atoms), confirming that the structure of the BRCT domain is not perturbed by the K543A mutation (Fig. 2D).

We next tested the importance of the KKT4 BRCT domain in vivo by expressing an RNAi-resistant form of KKT4 that has either the K543A mutation or T493A/T494A mutations fused with C-terminal YFP. The mutations did not affect the kinetochore localisation of full-length KKT4 (Fig. 5B). Upon induction of RNAi that depletes the untagged copy of KKT4, both mutant cell lines displayed severe growth defects (Fig. 5C), showing that a functional BRCT domain is essential for the proliferation of trypanosome cells.

## Conclusion

These NMR resonance assignments complement the previously reported X-ray structure of the BRCT domain (KKT4^463–645^) from *T. brucei.* We have confirmed that in solution the BRCT domain binds phosphate ion in the sulfate ion binding site previously identified by X-ray crystallography. This study will serve as a starting point for future experiments to probe in detail the interaction with phosphopeptides. The in vivo importance of phosphopeptide binding also demonstrates the potential for rational drug design to target neglected tropical diseases caused by kinetoplastid parasites.

### Supplementary Information

Below is the link to the electronic supplementary material.

Supplementary file1 (PDF 271 KB)

## Data Availability

Assignments for the KKT4 BRCT domain have been deposited in the BMRB under accession number 51542. The X-ray structure of the K543A variant of the KKT4 BRCT domain has been deposited in the RCSB Protein Data Bank under accession number 7QRO. The plasmids used in this study are available upon request.
