# Supplementary material for: NMR study of the structure and dynamics of the BRCT domain from the kinetochore protein KKT4
Source: Biomol NMR Assign. 2024 Mar 7;18(1):15–25. doi: 10.1007/s12104-024-10163-9 (PMC11081923; doi:10.1007/s12104-024-10163-9)
Supplement: Supplementary file 1 — Supplementary file1 (PDF 271 KB) [file 12104_2024_10163_MOESM1_ESM.pdf]

Supporting Table 1. Data collection and refinement statistics for the crystal structure of KKT4$^{463–645}$ K543A

**Data collection**	
Beamline	Diamond Light Source I03
Wavelength (Å)	0.9763
Space group (Z)	*P* $2_1\,2_1\,2_1$
Unit cell	46.66Å 61.34Å 67.75Å<br>90° 90° 90°
Resolution range (Å)	37.14—1.80 (1.86—1.80)
Unique reflections	18590 (1818)
Completeness (%)	99.95 (99.89)
Multiplicity	12.0 (1.2)
I/σI	89.9 (0.2)
R_{meas}	0.030 (1.580)
$CC_{1/2}$	1.0 (0.6)
Wilson B-factor (Å^2)	22.17
**Refinement**	
R_{work}	0.199 (0.197)
R_{free}	0.249 (0.257)
Number of atoms	1370
Protein	1180
Solvent	190
RMS bonds (Å)	0.007
RMS angles (°)	0.96
Ramachandran favoured (%)	96.60
Ramachandran allowed(%)	3.40
Ramachandran outliers (%)	0.00
Average B-factor (Å^2)	26.79
*Statistics for the highest-resolution shell are shown in parentheses.	

Supporting Table 2. Primers used in this study.

Name	Description
BA1363	GATCTTAATTAAGACGGATGAATACGTCAAAAAAG
BA856	GATCGGCGCGCCCTAATCACGACTTATAGCGAAAC
BA1732	CCGTGGTCCCCCACGGAGCGTGGCAGCGTTGTGCGGTGTTGTGTCCTCAAAATGG
BA1733	GGACACAACACCGCACAACGCTGCCACGCTCCGTGGGGGACCACGGAGAAC
BA2300	GCAGCCACGTTACTTTATTGCGGCGAGTCTAACAGAAAAGGAACGGAACAG
BA2301	CGTTCCTTTTCTGTTAGACTCGCCGCAATAAAGTAACGTGGCTGCACTGCCC

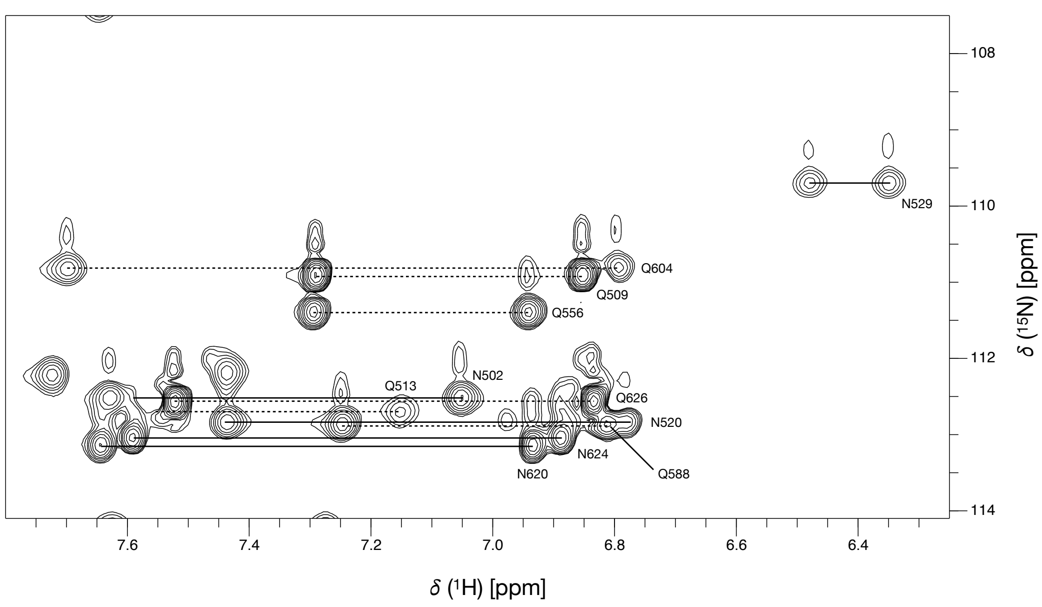

Supporting Figure 1

Part of the 750 MHz ^{1}H-^{15}N HSQC spectrum of $KKT4^{463–645}$ in 50 mM sodium phosphate, 100 mM NaCl and 0.5 mM TCEP (95% H_2O/5% D_2O), at pH 7.0, 20°C. The peak assignments for Asn/Gln amide side chains of $KKT4^{463–645}$ are labeled (the side chain of Q487 has not been assigned).

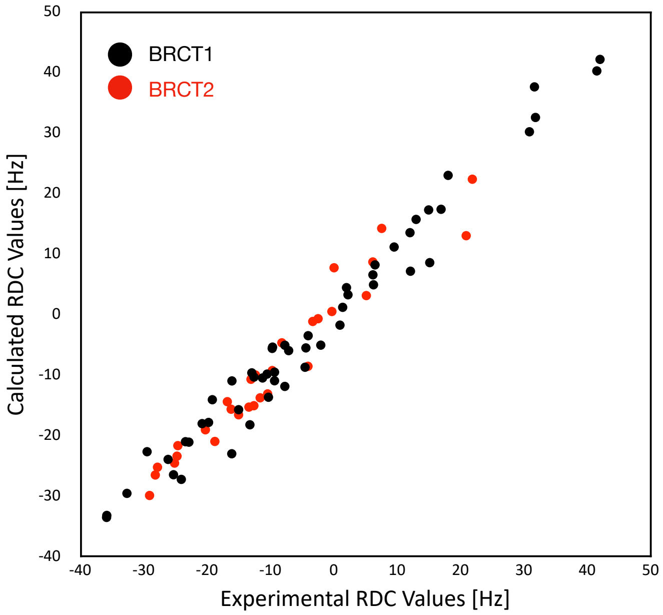

Supporting Figure 2

Plot of the experimental RDCs versus the RDCs calculated with a single D_a and R value but slightly different orientations of the alignment tensor for the 81 residues used in the fitting process. Residues in BRCT1 and BRCT2 are shown in black and red, respectively. The good agreement between experimental and calculated RDCs is shown by the Q value of 0.18.
